# Isoform-Specific Roles of Mutant p63 in Human Diseases

**DOI:** 10.3390/cancers13030536

**Published:** 2021-01-31

**Authors:** Christian Osterburg, Susanne Osterburg, Huiqing Zhou, Caterina Missero, Volker Dötsch

**Affiliations:** 1 Institute of Biophysical Chemistry and Center for Biomolecular Magnetic Resonance, Goethe University, 60438 Frankfurt, Germany; osterburg@bpc.uni-frankfurt.de (C.O.); s.osterburg@bpc.uni-frankfurt.de (S.O.); 2 Radboud Institute for Molecular Life Sciences, Molecular Developmental Biology, Radboud University, 6525 GA Nijmegen, The Netherlands; j.zhou@science.ru.nl; 3 Human Genetics, Radboud Institute for Molecular Life Sciences, Radboud University Medical Center, 6525 GA Nijmegen, The Netherlands; 4 Department of Biology, University of Naples Federico II, 80126 Napoli, Italy; missero@ceinge.unina.it; 5 Center for Genetic Engineering, CEINGE Biotecnologie Avanzate, 80145 Napoli, Italy

**Keywords:** p63, EEC syndrome, AEC syndrome, stratified epithelial tissues, enhancer, chromatin remodeling, transcriptional regulation, fertility, epidermis, aggregation

## Abstract

**Simple Summary:**

The protein p63 belongs to the family of the p53 tumor suppressor. Mouse models have, however, shown that it is not a classical tumor suppressor but instead involved in developmental processes. Mutations in the *p63* gene cause several developmental defects in human patients characterized by limb deformation, cleft lip/palate, and ectodermal dysplasia due to p63’s role as a master regulator of epidermal development. In addition, p63 plays a key role as a quality control factor in oocytes and p63 mutations can result either in compromised genetic quality control or premature cell death of all oocytes.

**Abstract:**

The p63 gene encodes a master regulator of epidermal commitment, development, and differentiation. Heterozygous mutations in the DNA binding domain cause Ectrodactyly, Ectodermal Dysplasia, characterized by limb deformation, cleft lip/palate, and ectodermal dysplasia while mutations in in the C-terminal domain of the α-isoform cause Ankyloblepharon-Ectodermal defects-Cleft lip/palate (AEC) syndrome, a life-threatening disorder characterized by skin fragility, severe, long-lasting skin erosions, and cleft lip/palate. The molecular disease mechanisms of these syndromes have recently become elucidated and have enhanced our understanding of the role of p63 in epidermal development. Here we review the molecular cause and functional consequences of these p63-mutations for skin development and discuss the consequences of p63 mutations for female fertility.

## 1. Introduction

Failure to maintain cellular homeostasis is a common driver of diseases like cancer and neurodegeneration, and also plays a role in ageing, immunological, and metabolic disorders. Quality control systems are the first line of defense that detect potentially stressful or dangerous conditions and provide the information for the cell to initiate countermeasures. Similarly, proper response to environmental cues and stress controls correct cell fate decisions that are essential for development and homeostasis of tissues and organs. Depending on the type of stress, the cellular response can include, for example, activation of DNA repair, cell cycle arrest, expression of chaperones, inhibition of translation, or activation of an inflammatory response. If the stress cannot be resolved, cell death pathways can become activated. One of the most important integrators of cellular stress response pathways is the tumor suppressor p53 [1]. DNA damage, nucleotide imbalance, as well as activation of oncogenes and other stress pathways stabilize p53, which under normal conditions is kept at low concentration in unstressed cells by the action of E3 ligases, in particular Mdm2 and Mdm4 [2,3,4]. Stabilization of p53 triggers its binding to promotor regions of certain genes, which results in the transcription of genes involved in DNA repair, senescence, cell cycle arrest, and apoptosis [5,6,7,8]. Depending on the type of stress and the severity of the damage cells can either temporarily get arrested in their cell cycle or permanently (senescence). If the damage is too severe, an apoptotic program gets initiated [9,10]. This central surveillance function makes p53 the most important tumor suppressor as well as the most often mutated gene in tumor cells [11,12], and has earned p53 the nickname “guardian of the genome” [13].

The discovery that p53 has two siblings—called p63 [14] and p73 [15]—originally sparked speculations that tumor suppression is orchestrated by the interplay of all three family members. Inactivation of either gene in mice, however, showed strikingly different phenotypes from each other as well as from the p53^−/−^ mouse. The p73^−/−^ mouse suffers from abnormal hippocampal development, hydrocephalus, chronic infections and inflammation, as well as abnormalities in pheromone sensory pathways [16]. The phenotype of the p63^−/−^ mouse is even more severe. It is born alive, but dies soon after birth caused by multiple developmental defects that include limb truncations and the lack of a multilayered skin and other epithelial structures. These mouse models are in stark contrast to the p53^−/−^ mouse which shows mostly relatively mild developmental defects such as ocular abnormalities and defects in tooth formation as well as death of ~20% of female embryos and newborn mice due to defects in neural tube closure [17,18]. These mouse models have clearly demonstrated the importance of p63 and p73 in developmental processes. Further detailed investigations showed that p73 is involved in regulating cell–cell contacts between developing sperm cells and Sertoli cells which are important for sperm cell maturation [19,20]. Male p73^−/−^ mice are infertile due to lack of mature sperm cells. In addition, p73 is essential for the development of multiciliated cells as, for example, present in the trachea [21]. Lack of these cells explains the observed chronic infections of p73^−/−^ mice [16], which is caused by an inefficient mucus transport and clearance of foreign material including pathogens from the airways.

For p63, two different main functions have been identified: p63 is responsible for the development and maintenance of stratified epithelial tissues such as skin [22,23,24], and it is the main quality control factor that monitors the genetic integrity in oocytes [25,26]. This quality control function seems to be the evolutionary oldest function of the entire p53 protein family, as even evolutionary distant species such as *Caenorhabditis elegans* [27,28], *Drosophila melanogaster* [29,30], *Ciona intestinalis* [31], or sea anemones [32] express a protein in their germ cells that resembles p63 [33]. In mammals, the genetic quality control is carried out by a specific isoform, called TAp63α [25,34]. This isoform gets expressed at the end of the process of homologous recombination when oocytes have repaired the DNA double strand breaks inflicted by the type II topoisomerase-like DNA transesterase Spo11 [35,36,37]. If DNA double strand breaks are still present TAp63α initiates an apoptotic program via the expression of PUMA and NOXA [38] that eliminates damaged oocytes. Following this check point oocytes enter dictyate arrest and remain arrested in prophase of meiosis I until they get recruited for ovulation. During the entire time—which in humans can last for ~50 years—TAp63α is highly expressed. To prevent the initiation of apoptosis in non-damaged oocytes, TAp63α adopts a closed, inactive, and only dimeric conformation [39,40]. Detection of DNA damage results in the phosphorylation of S582 of TAp63α by the kinase Chk2 [41]. While this modification does not activate TAp63α, it recruits another kinase, CK1 [42], which in general requires pre-phosphorylated substrates. The addition of four more phosphate groups switches TAp63α to its active and tetrameric conformation [42]. This activation is irreversible [40,43] and de-phosphorylation does not convert the protein back to its inactive dimeric state. Oocyte death resulting in Premature Ovarian Insufficiency (POI) can be triggered, for example, by chemotherapy and irradiation [44,45,46,47,48,49] as used for the treatment of cancer patients or by mutations in p63 [50] that abrogate the function of the C-terminal Transactivation inhibitory domain (TID) [51] (see also Section 5 below).

The developmental function of p63 in epithelial tissues is based on a different isoform, ΔNp63α. The importance of this isoform for epidermal development was further shown by a knockout mouse model with specific inactivation of the ΔNp63 isoforms, which shows the same epithelial defects as the p63^−/−^ mouse [52] as well as by the rescue of the p63^−/−^ phenotype by expressing ΔNp63α under the K5 promoter [53]. ΔNp63 is generated by activation of an alternative promoter located in intron 3. ΔNp63α lacks the N-terminal 69 amino acids that include the transactivation domain, which in p63 is contained exclusively in the first 24 amino acids [54] that form a single helix (in contrast to the bipartite transactivation domains found in p53 [55] and p73 [56]). Instead, ΔNp63α has 14 unique amino acids at its N-terminus [14]. While ΔNp63α is basically inactive as a transcriptional activator on promoter sequences that are typically also bound by p53 (such as the p21 or Mdm2 promoters) [14], RNAseq (RNA sequencing), and ChIPseq (Chromatin ImmunoPrecipitation DNA-Sequencing) studies have revealed that this isoform is responsible for an epithelial-specific transcriptional program [57,58,59] as well as organizing the chromatin structure [60,61,62,63]. Recent progress in our understanding of the function of this isoform in the development of epithelia and attached appendages has also increased our knowledge how mutations in p63 result in different human syndromes and affect female fertility. This progress will be reviewed here.

## 2. P63 in the Development of Stratified Epithelial Tissues

The analysis of the p63^−/−^ mouse revealed that it suffers from a lack of stratified epithelial tissues [22,23]. The skin as the largest organ is composed of several cell layers. The basal layer cells are attached to the basement membrane separating the epidermis from the dermis and actively proliferate maintaining tissue homeostasis. When these progenitor cells detach from the basement membrane, they cease to proliferate and move in an upward path of differentiation [64,65] that includes several discrete transcriptional stages as they form the spinous and granular layers before they terminally differentiate into cornified cells that form the outer most skin layer. As these dead cells are eventually shed, they have to be renewed continuously. For this tissue homeostasis, a functional stem cell compartment is essential. The p63^−/−^ mouse has demonstrated that the maintenance of this compartment is dependent on the expression of p63 with its homozygous inactivation resulting in a disorganized pseudostratified epithelium that does not undergo full differentiation and expresses simple epithelial markers such as keratin K8/K18. Wild type skin shows a clear gradient of p63 expression with high expression in the basal layer followed by decreasing expression in more differentiated keratinocytes and loss of expression in the upper layers [14]. In addition to skin, other stratified epithelia express p63 in their basal compartment and consequently are not fully developed in the p63^−/−^ mouse. These tissues include esophagus, the proximal portion of the stomach, cervix, urogenital tract, prostate, and breast, as well as structures derived from the same stem cells responsible for these tissues such as mammary, sebaceous, lachrymal, and salivary glands [22,23]. Furthermore, transient epithelial structures during development are also affected in the p63^−/−^ mouse, such as the apical ectodermal ridge (AER) on the limb buds, a region of specialized, multilayered epithelium, which is essential for the development of distal limbs, digit patterning, and morphogenesis [66,67], and defects in AER lead to limb abnormalities.

In addition to these squamous stratified epithelial tissues, p63 is expressed also in pseudo-stratified epithelia such as the epithelium of the trachea where p63 is found in the basal cell layer [21]. Interestingly, ~50% of these cells also show expression of p73. Since p63 and p73 preferentially form 2:2 hetero-tetramers [68,69,70], transcriptional regulation in these cells, as well as in those cells of the epidermis that express both proteins, might depend on hetero-tetramers formed by combination of different p63 and p73 isoforms. While currently the role of p73 in skin development has not been fully clarified with the p73^−/−^ mouse not showing any obvious epidermal defects [16], first results have demonstrated that a lack of p73 influences wound healing processes in mice [71].

The important role of p63 for the integrity of stratified epithelial tissues is tightly associated with its regulatory role in gene expression. Consistent with in vivo observations, cellular studies have shown essential roles of p63 in cell proliferation, differentiation, and cell adhesion [58,72,73] in epidermal keratinocytes. These processes are compromised in p63 knock-down cells, as evident also from impairment of differentiation and stratification in 2D and 3D models [59,74]. This defect is caused by reduced expression of proteins involved in keratinocyte differentiation programs, with several of them being direct transcriptional targets of ΔNp63α such as K14 [75], Claudin1 [76], or Perp [77], as shown by classical molecular and cellular approaches. Recent transcriptomic and epigenomic technologies have provided overwhelming evidence that confirms previous studies (reviewed in [78]), and further scrutinizes a master regulatory role of p63 in epidermal commitment and development. Up to now, p63 has been shown to regulate gene expression at three levels: By controlling the enhancers to regulate specific gene expression, by acting as a pioneer factor through opening chromatin and recruiting other transcription factors to establish proper chromatin environments for gene expression, and by cooperating with chromatin modifiers to regulate transcription in a higher-order. These regulatory mechanisms are not mutually exclusive.

### 2.1. Binding of p63 to Enhancer Elements

Different from previous expectation that p63 regulates only a few dozens of direct targets, tens of thousands of p63 binding sites [79] have been reported in human and mouse keratinocytes by a number of p63 ChIP-seq studies [61,62,80,81,82,83]. Another surprise of these unbiased genome-wide studies is that p63 mainly binds to non-coding genomic regions, potentially enhancers, rather than gene promoters. The important role of p63 in controlling epidermal enhancers is further underscored by several recent ATAC-seq analyses that identified enhancers in the genome [84,85,86,87]: The p63 binding motif is one of the most significantly enriched transcription factor binding motifs in identified enhancers in keratinocytes, consistent with its master regulatory role. Similarly, in a genome wide study of enhancer elements in keratinocyte progenitors and differentiated keratinocytes p63 binding was found to be enriched in super enhancer elements [88]. This is consistent with a study showing that in clustered epidermal-specific enhancers (clustered H3K27ac-marked enhancers that are important for cell-type gene expression [89,90]), p63 binding sites are enriched, with three binding sites per enhancer on average [61]. Furthermore, p63 was also found to associate with Dnmt3a to maintain high levels of DNA hydroxymethylation at the center of enhancers in a Tet2-dependent manner [91]. Interestingly, it seems that not all p63 binding sites are active enhancers. This was shown by a study where ChIP-seq analysis of p63 and histone H3K27ac, a modification that marks active enhancers, was performed, and p63 binding sites do not always co-localize with H3K27ac [61]. This study also demonstrated that gene expression activity does not correlate with p63 binding itself, but with p63 binding to active enhancer regions. These observations lead to the interpretation that p63 may function as a bookmarking factor in the genome during epithelial lineage commitment, and inactive chromatin regions bound by p63 can become active during the following stages of development/differentiation. By binding to enhancers, p63 may recruit other transcription factors, co-activators (e.g., p300/CBP) or repressors (e.g., HDACs) to fine tune gene expression. This is consistent with models that p63 can function as both an activator and a repressor [85] to regulate specific gene expression.

### 2.2. Cooperation with Other Transcription Factors

To coordinate specific transcriptional programs, p63 cooperates with other transcription factors. Cooperation with TFAP2C was observed in a study using ATACseq (Assay for Transposase-Accessible Chromatin using sequencing) experiments and transcriptome data from hESCs undergoing epidermal differentiation [92]. In this investigation, TFAP2C acts as the ‘‘initiation factor’’, for a surface ectoderm differentiation program. Subsequently, p63 functions as the ‘‘maturation factor’’, that modifies the prepatterned chromatin landscape into that of functional keratinocytes. Cooperation of p63 with both TFAP2A and TFAP2C was also seen in ChIPseq analysis of primary human neonatal foreskin keratinocytes. Depletion of either of these two AP2 transcription factors interferes with terminal differentiation in organotypic epidermal skin cells [81]. Other transcription factors that likely cooperate with p63 were identified by analysis of ChIPseq experiments and include AP-1, bZIP, RUNX, ZFX, and EBF1 transcription factor family members [61,80,93,94]. The importance of p63 in regulating epithelial cell specific transcriptional programs was also demonstrated in transdifferentiation experiments. Enforced expression of ΔNp63 and KLF4 in differentiated fibroblasts induces the conversion into keratinocyte-like cells expressing keratinocyte markers such as K14, but lacking typical fibroblast markers including MME and COL11A1 [95,96]. Similarly, the combination of ΔNp63 and PAX6 was shown to be essential for the epithelial fate of limbal stem cells in the cornea [97,98,99].

### 2.3. Role of p63 for Chromatin Remodeling

The combination of several investigations has shown that p63 is involved in higher order regulatory mechanisms by cooperation with chromatin remodeling proteins. Among these proteins are SATB1 [100], a protein important for remodeling the chromatin architecture at the epidermal differentiation complex (EDC) gene locus (a chromosomal region rich in genes required for terminal epidermal differentiation) and CBX4, a component of the Polycomb Repressive Complex 1 [101] that is required for repressing nonepidermal gene expression. Enhanced expression of either of these two remodelers can partially rescue p63 deficiency in keratinocytes. Further epigenetic reprogramming factors under p63 transcriptional control are BRG1 [102] and LSH [103] as well as certain components of the nuclear envelope [82], which plays an important role in the spatial organization of overall transcriptional activity and provides a mechanistic explanation for the observed nuclear shape of skin cells in p63^−/−^ mice. Direct interaction of p63 with epigenetic factors has also been observed in the case of HDAC1/2 which bind to the C-terminal TI domain [83,85]. This domain is used to keep TAp63α in oocytes in its inactive, only dimeric conformation [39,51,104], but is freely accessible in the constitutive open and tetrameric state of ΔNp63α. Furthermore, cooperation between p63 and the genome organizer CTCF has been observed, which may not be based on direct protein–protein interaction, but on binding to enhancer regions in proximity [84].

In line with the already mentioned bookmarking role of p63 in binding to enhancers, a pioneer function role of p63 has also been proposed. Comparison of loci to which p63 is bound in keratinocytes with the same loci in other cell types has demonstrated that without endogenous p63 expression these loci are nucleosome-enriched, compact, and hence transcriptionally inactive [105]. In keratinocytes, p63 and the ATP-dependent chromatin remodeling BAF complex (SWI/SNF in yeast) mutually recruit each other to DNA sequences to remodel the chromatin structure and to recruit the transcriptional machinery to sites important for cell-type specific gene expression [106]. However, these studies using keratinocytes have only provided indirect evidence for the pioneer function of p63. This is because, by definition, a pioneer factor can bind to nucleosome rich regions and open the chromatin to regulate gene transcription. In keratinocytes enhancer regions bound by p63 are accessible, while p63 binding to closed chromatin regions cannot be detected. One recent study in zebrafish development has, however, convincingly demonstrated that p63 binds to nucleosomal closed chromatin regions at an early stage of embryogenesis before the epidermal fate is established, and these regions become open and bound by p63 in cells with epithelial fate [107].

In summary, the data revealed by novel epigenomic technologies have consolidated the master regulatory role of p63 in stratified epithelia.

## 3. Syndromes Caused by Mutations in p63 in Human Patients

**Figure 1:**
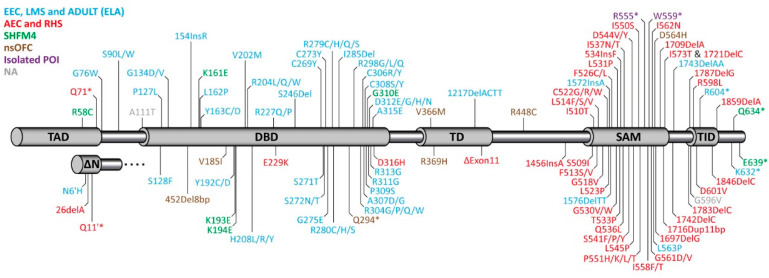
Domain structure of TAp63α and ΔNp63α and distribution of mutations identified in different syndromes of human patients. TAD: Transactivation domain; DBD: DNA binding domain; TD: Tetramerization domain; SAM: Sterile alpha motif domain; TID: Transactivation inhibitory domain. Mutations causing the EEC (Ectrodactyly-ectodermal dysplasia-clefting), LMS (Limb mammary syndrome), and ADULT (Acro-Dermato-Ungual-Lacrimal-Tooth malformations) syndromes (light blue) are mainly found in the DBD, mutations causing the AEC (Ankyloblepharon-ectodermal defects-cleft lip/palate), RHS (Rapp-Hodgkin syndrome) syndromes, or isolated premature ovarian insufficiency (POI) cluster in the C-terminus (SAM and TID). Mutations causing SHFM4 (split-hand/split-foot malformations) or nsOFC (non-syndromic orofacial clefting) are found throughout the sequence. NA (grey color) summarizes mutations that cannot be assigned to any of the above mentioned disorders.

The publication of the phenotype of the p63^−/−^ mouse led to the identification of mutations in p63 being responsible for several human syndromes [108,109,110] (Figure 1). Originally, five different syndromes could be correlated with mutations in different domains of p63 [111,112]. Point mutations in the DNA binding domain result in the Ectrodactyly-ectodermal dysplasia-clefting (EEC) syndrome (OMIM 604292), which is characterized by a combination of (1) limb deformation, (2) cleft lip/palate, and (3) ectodermal dysplasia. EEC patients also suffer from a severe corneal limbal stem cell deficiency (LSCD) [98], which can lead to progressive vision loss at young adult age. The role of these mutations was confirmed in heterozygous mouse models expressing EEC mutations, which show defects resembling the human phenotype [113]. Most of the reported mutations affect five hot spots located at amino acids R204, R227, R279, R280, and R304 that are all involved in the DNA-protein interface. Genotype–phenotype analysis, however, has revealed that mutations of these residues affect patients differently, and the effects depend on the exact amino acid change [112]. For example, the R304 mutation is connected to a high likelihood of developing cleft lip/palate, while patients with the R227 mutation only rarely show this defect. Similarly, syndactyly is absent in these patients, while it has a 30–60% prevalence in patients with the other hot spot mutations. In contrast, genito-urinary defects are seen significantly more often in patients with R227 mutations than in patients with mutations of the other hot spot residues [112]. In addition to missense mutations in the DNA binding domain, the EEC syndrome can also be caused by frame shift mutations in the C-terminus of the protein (Figure 1).

Mutations responsible for the Ankyloblepharon-ectodermal defects-cleft lip/palate (AEC, OMIM 106260) syndrome are found in the C-terminus of p63 [110] including the Sterile alpha Motif (SAM) domain and the transactivation inhibitory domain (TID, Figure 1). Most patients suffering from AEC syndrome show severe skin erosions at or after birth that can be life threatening and can last for long time. Approximately, 45% of patients show ankyloblepharon (closed eye lids) and defects in nail, teeth, and other epidermal appendages are more present than in other p63-related syndromes. Other defects include genito-urinary defects, hearing impairment, cleft lip, and cleft palate. Limb defects, however, are rarely reported (mild syndactyly) and ectrodactyly is absent. Only preventive and palliative care is provided for this serious illness as there are no approved therapeutic options.

Other p63-related syndromes—Limb mammary syndrome (LMS, OMIM 603543), Acro-Dermato-Ungual-Lacrimal-Tooth malformations syndrome (ADULT, OMIM 103285), and Rapp-Hodgkin syndrome (RHS, OMIM 129400) are similar to either the EEC (LMS, ADULT; also summarized as the ELA syndrome [114]) or AEC (RSH) syndrome [115], respectively. The two non-syndromic disorders isolated split hand/foot malformation [116] (SHFM4, OMIM 605289) and non-syndromic cleft lip [117] (OFC8, OMIM 129400) have a less well defined genetic basis as mutations connected to these disorders are spread throughout the p63 gene.

Mutations in p63 that cause the syndromes mentioned above are believed to act via a dominant negative or gain of function mechanism. Haploinsufficiency was thought not to be relevant for p63-caused syndromes. This interpretation was mainly based on the observation that individuals who have a constitutive terminal deletion of the long arm of chromosome 3, which harbors the p63 gene locus do not suffer from EEC like syndromes [118]. This was further supported by knockout mouse studies showing that mice with a heterozygous *TP63* deletion develop normally [22,23]. In contrast to these earlier observations the recent identification of patients with premature stop codons in the DBD or missense mutations in the OD showing orofacial clefting suggests that also gene dosage can affect p63-dependent developmental programs in humans [119]. The two stop codons identified in the DBD likely result in degradation of the mRNA via nonsense-mediated decay, while for at least one of the missense mutations within the OD it could be shown that it destabilizes the tetrameric state. These results show that loss of function of p63 is a risk factor for orofacial clefting. An overview of the most common phenotylic characteristics of patients suffering from syndromes caused by mutations in p63 is given in Table 1.

## 4. Molecular Disease Mechanism

### 4.1. EEC Syndrome

**Figure 2:**
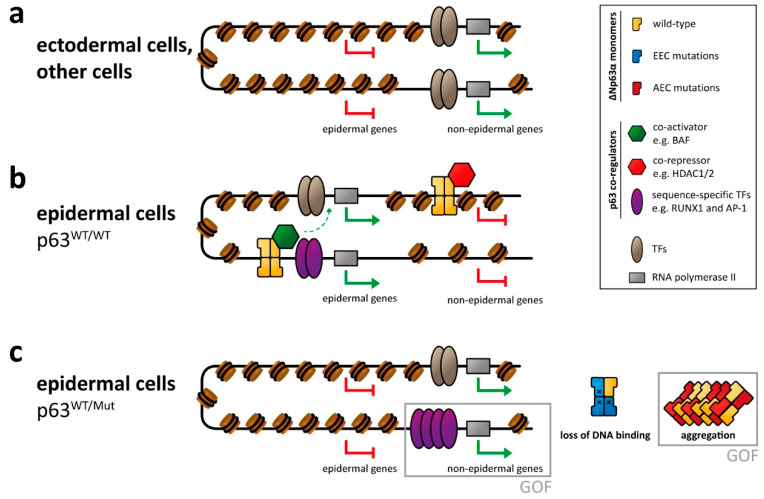
p63-mediated gene regulation affected by syndromic mutations. (**a**) In ectodermal and other non-epidermal cell types, the chromatin of enhancer and promotor regions of epidermal genes is not accessible, resulting in repression of those genes. (**b**) In epidermal cells p63 binds to promotor and (super-)enhancer regions of epidermal genes and cooperates with other transcription factors (TFs) and epigenetic modifiers as co-activators in transcribing those genes. Transcription of non-epidermal genes is repressed by the recruitment of epigenetic modifiers acting as co-repressors. Other non-epidermal genes are repressed indirectly (due to lack of non-epithelial TFs or inaccessible chromatin). (**c**) In cells with heterozygous p63 mutations, the proper p63-mediated chromatin and transcriptional regulation is affected, resulting in silencing of epidermal genes as well as the expression of non-epidermal genes. This is caused either by loss of p63-related repression or by a gain of function (GOF) mechanism via transcriptional activation by overexpressed/deregulated transcription factors normally associated with p63. In case of the EEC syndrome, this GOF mechanism has been demonstrated for the RUNX1 transcription factor. While not formally demonstrated yet, GOF effects are also predicted for the AEC syndrome via a co-aggregation mechanism.

The Ectrodactyly, Ectodermal Dysplasia, and Cleft lip/palate syndrome is the most common and best investigated p63-related syndrome. Biochemical studies have revealed that the missense mutations in the DNA binding domain result in loss of DNA binding capability [108], which due to the formation of tetramers through the oligomerization domain exhibits a dominant negative effect in the typical heterozygous patient situation (Figure 2). This dominant negative effect of p63 EEC mutants on p63 binding was confirmed by a recent ChIP-seq study using EEC patient keratinocytes [62]. In addition, mutant p63 shows an increased half-life in patients as well as in cell culture [123], which leads to an accumulation of the mutated protein. Through mutational analysis in cell culture and the use of siRNA to decrease the p63 level it was shown that p63 knock down leads to proliferation defects resulting in hypoplasia [59]. Mechanistically, knock-down of ΔNp63α or loss of DNA binding due to mutation increases expression of cell cycle inhibitors such as p21 [86] and a decreased expression of positive regulators such as Fos and c-Jun [124].

Due to the master regulator function of p63, loss of DNA binding by EEC-type mutations has drastic effects on the chromatin landscape and transcriptome. Through a combination of ChIPseq and ATAC-seq analysis, it was shown that the entire enhancer landscape is changed in EEC patient keratinocytes [61,62,84]. While loss of active enhancers bound by p63 is expected, surprisingly, other active enhancer elements appeared. These new active enhancers are often bound by the transcription factor RUNX1 (as a gain of function effect of EEC mutations). Down regulation of RUNX1 in mutant p63 cells could partially rescue this effect. Similar to the developmental defects reported in p63 knock-down keratinocytes [59], EEC patient keratinocytes bearing either the R204W, the R304W, or the R279H p63 mutation showed deregulated gene expression in combination with a rewired enhancer landscape resulting in differentiation defects in 2D and 3D culture [125]. Investigating the effect of EEC mutations (R204W and R304W) in human induced pluripotent stem cells during differentiation into keratinocytes showed defects in epidermal commitment [63]. In particular the transition from the simple epithelium to the basal stratified epithelium was blocked in cells bearing EEC mutations as identified via transcriptome analysis. This analysis also identified mesodermal genes being upregulated in the EEC mutant cells. The use of inhibitors of mesodermal activation resulted in a partial rescue of the transcriptome indicating an enhanced epidermal commitment [63].

Investigation of the defects in limb formation has revealed that Dlx5 and Dlx6 are direct targets of ΔNp63α [80,126]. Both belong to the group of distal-less-related homeodomain transcription factors that are essential for the development of head and limb skeleton [127,128]. ΔNp63α as well as Dlx5 and Dlx6 are expressed in the apical ectodermal ridge (AER) of the limb buds [22,23,129]. Similar results with defects of the formation of the AER by loss of p63 have been reported in zebrafish where the AER is responsible for fin formation and ectodermal derivatives [130].

### 4.2. AEC Syndrome

The Ankyloblepharon Ectodermal defects-Cleft lip/palate (AEC) syndrome is caused by three different types of genetic variations in the p63 gene: (1) Point mutations in the SAM domain, (2) point mutations within the TI domain, and (3) frame shift mutations C-terminal to the oligomerization domain [110]. To gain further insight into the disease mechanism of AEC-causing missense mutations, a knock-in mouse harboring a L514F mutation (p63^+/L514F^), known from human patients to cause AEC, was created and analyzed [131]. This mutation acts as a dominant negative resulting in a defective stem cell compartment that leads to severe epidermal hypoplasia. Analysis of differentiation markers, however, showed that no significant difference to wild type keratinocytes could be detected. This led to the interpretation that the L514F mutation in the SAM domain results in a transient reduction in cell proliferation during epidermal development, but no overt defects in terminal differentiation. While no difference could be detected in the expression level of typical p63 controlled cell cycle genes (p21, p16, p19, or miR-34a) or in keratinocyte differentiation markers, FGF signaling components were markedly reduced in p63^+/L514F^ epidermis and palate. In particular, the mRNA level of the epidermal specific isoforms Fgfr2b and Fgfr3b were reduced, while the levels of Fgfr1 and Fgfr4 did not show differences relative to wild type or p63^+/−^ tissues. These results are consistent with the identification of p63 binding sites in Fgfr2 and Fgfr3 loci by ChIPseq. Epidermal cells self-renewing potential was affected both in p63^+/L514F^ and in Fgfr2b^−/−^ keratinocytes. Interestingly, the Fgfr2b^−/−^ mouse shows epidermal hypoplasia, tooth and hair defects, as well as cleft palate, strongly phenocopying the AEC syndrome.

Other transcriptional p63 target genes that were found downregulated by AEC mutations, at least in human cells exogenously expressing AEC mutant p63, are HOPX, GRHL3, KLF4, PRDM1, and ZNF750 [132,133], transcriptional regulators required for epidermal differentiation. Restoring expression of ZNF750 in these model tissue resulted in rescue of epidermal differentiation. The skin fragility observed in AEC patients could be explained at least in part by microscopic blistering among basal cells and between basal and suprabasal compartments caused by reduced expression of desmosomal cadherins and desmoplakin, which are direct p63 target genes [73]. Interestingly, treatment with the Epidermal Growth Factor Receptor (EGFR) inhibitor Tyrphostin AG1478 resulted in increased mechanical resistance of keratinocytes with a L514F mutation or siRNA based p63 knockdown [73] in agreement with results from epithelial cancer cells [134,135].

To understand the structural consequences of the L514F mutation in the SAM domain, the structure of the mutant form was determined by NMR spectroscopy and compared with the structure of the wild type domain [96]. No significant differences could be seen between the structures, the only difference being a slightly looser packing of the hydrophobic core of the mutant. This results in a destabilization of the SAM domain with the melting temperature reduced from 78 °C to 65 °C [96], however, still much higher than the 37 °C body temperature. The decisive difference between the wild type and the mutant domain is that the unfolded wild type domain refolds upon cooling while the mutant domain aggregates and precipitates [96]. Sequence analysis showed that the SAM domain contains two sequence stretches with high aggregation propensity. These stretches are usually inaccessible because they are located in helices 1 and 3 of the folded structure. Destabilization and—probably a slower kinetics of folding—result in the exposure of these sequences, which triggers aggregation of p63 [96] (Figure 2). This aggregation behavior could be confirmed in vitro, in cell culture, as well as in cell extracts obtained from the epidermis of a conditional p63^+/L514F^ mouse model [96]. Analysis of other mutations in the SAM domain revealed the same destabilization, which in some cases (G530V, I537T) can be dramatic and result in the inability of the domain to fold at body temperature at all [136]. Interestingly, sequence analysis of the point mutations in the TID of p63 and frame shift mutations show that in each case, aggregation prone peptides are being formed (missense mutations) or newly created (frameshift mutations). Variants of p63 that abolish aggregation of the mutant proteins are able to rescue its transcriptional function and restore fibroblast-to-keratinocyte conversion [96]. Aggregation of p63 is, thus, the unifying molecular mechanism of all mutations causing the AEC syndrome and mechanistically clearly distinguishes the AEC syndrome from the EEC syndrome (Figure 2).

## 5. Impact of p63 Mutations on Female Fertility

**Figure 3:**
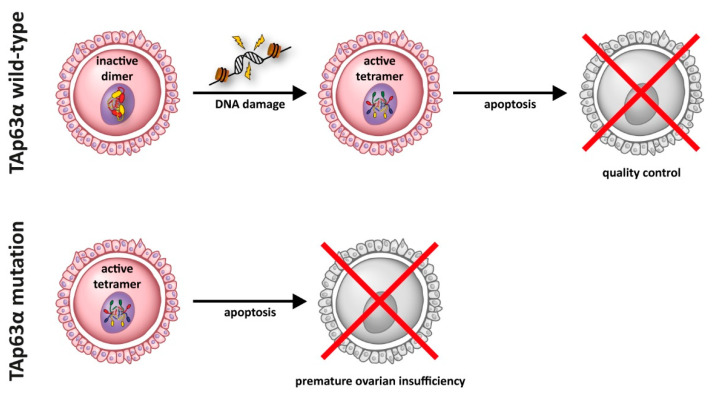
In resting oocytes, TAp63α is kept in an inactive state as a closed dimer. This dimer is converted into an active tetrameric state through a kinase cascade following the detection of DNA damage. In its active tetramer form, TAp63α induces an apoptotic program in damaged oocytes. Certain mutations of TAp63α result in the formation of a constitutive active and tetrameric form that induces oocyte death even without DNA damage, thus resulting in premature ovarian insufficiency.

In addition to its role as master regulator of epidermal development, p63 is the master quality control factor in oocytes (“guardian of the female germ line”) [34,137,138]. In the germ cells, a different isoform of p63, TAp63α, which contains the full length N-terminal transactivation domain, is expressed. The importance of p63 for maintaining the genetic quality in oocytes was originally not discovered by the analysis of the p63^−/−^ mouse despite lacking functional p63 in the oocytes and required a later TA-isoform specific knock out mouse [25]. This original oversight of the oocyte function can be explained by the fact that ovaries lacking p63 appear normal with a high number of primary oocytes as typical around the time of birth in mice. Only upon detection of DNA damage—either due to natural sources such as unrepaired DNA double strand breaks (DSBs) following homologous recombination or external sources such as DSBs inflicted by ionizing radiation or chemotherapeutics—TAp63α becomes activated and eliminates oocytes, thus reducing their number [25,39,42]. P63 mutations seen in human patients do not only affect epidermal development, but also impact either the monitoring function of p63 and thus the quality of genetic surveillance or result in uncontrolled activity of p63 leading to premature exhaustion of the oocyte pool. While EEC mutations in the DBD of p63 will disable its ability to induce an apoptotic program in oocytes, EEC frame shift mutations that eliminate the TI domain create constitutively tetrameric and active p63 mutants that result in the rapid elimination of all oocytes. AEC missense mutations in the SAM domain will likely have no effect on the oocyte pool and will result either in closed dimeric conformations (if the SAM domain folds correctly) or in an inactive aggregate (if the SAM domain unfolds). Frame shift mutations and missense mutations in the TI domain can create open and in principle active tetramers, which however will have a reduced overall activity due to aggregation of the p63 mutants. If aggregation is slow compared to translation, formation of tetramers, and nuclear import, these mutants can result in a premature loss of the oocyte pool (Figure 3).

Some reports of the effect of p63 mutations on female fertility have started to appear in the literature. Nonsense mutations within the SAM domain (R555*, W559*) [139,140] have been reported in two young women suffering from premature ovarian insufficiency. Two frameshift mutations (delC1783) [122] and (delTT1576) [120] resulting in POI or even a complete absence of ovaries were reported as well as nonsense mutations in the TID of p63 [121]. In addition, POI patients with an intragenic duplication in *TP63* [141], leading to a nonfunctional TID and thus activated TAp63, were described.

## 6. Conclusions

The p53 protein family is arguably one of the most important protein families for genetic quality control and integration of stress and repair pathways in the cell. The p73 and p63 knock out mouse studies in combination with the identification of several developmental syndromes in human patients have shed a new light on the evolutionary origin of this family. Likely, the original function was that of a genetic quality control factor in germ cells, which are virtually immortal, as germ cells are also the source of the germ cells of the following generation. Strict quality control of the genetic integrity is, therefore, important for all organisms. In the case of p63, this control function of germ cells as the most important stem cell was then transformed into its role as a master regulator of epithelial development. Despite its high sequence similarity to p53, p63 does not seem to have a tumor suppressor activity [142], which is also supported by the observation that human patients with p63 mutations do not have a higher risk of developing cancer (although the number of patients is admittedly rather low). Instead, overexpression of ΔNp63α is reported in several squamous cell carcinoma (SCC) types, in particular head and neck SCC [143]. One effect of overexpression of ΔNp63α in these cells is that it blocks the activity of p53 and p73 [70,144]. The identification of p63 as a master regulator of epithelial development, however, makes it likely that its overexpression has additional disruptive effects on gene expression programs. The role of p63 in tumorigenesis has been well investigated in the case of breast cancer. p63 is expressed in the basal compartment of mammary glands that includes rare stem cells and myoepithelial cells [145,146]. The regulation of stemness, proliferation, and differentiation in these basal cells of the breast epithelium is particularly crucial as this tissue shows a high degree of expansion and involution during puberty, pregnancy, and breastfeeding suspension. Interestingly, in the regulation of expansion as well as apoptosis of cells during involution both TAp63α as well as ΔNp63α seem to be involved. Mice specifically lacking the TAp63 isoform develop metastatic mammary and lung adenocarcinoma [147], and TAp63α has been found in several breast cancer derived cell lines with mutations in the TA domain [148]. Recently, another isoform, called TA*p63α, was identified in Sum 159 cells [149]. This isoform also adopts a closed, only dimeric conformation that, through an N-terminal extension, is even more inhibited than TAp63α [14,149]. While clearly more research is required to understand the specific transcriptional programs regulated by different p63 isoforms, it seems likely that, in cells of the basal compartment of mammary glands, the interplay of ΔNp63α and TAp63α is crucial for normal tissue development and its misregulation contributes to the development of breast cancer. The current knowledge on the role of p63 in mammary gland and breast cancer development is presented in a recent excellent review [150]. p63′s role in regulating mammary gland biology is not restricted to the basal cells that express this transcription factor. It has been the discovered that the epidermal growth factor family ligand gene *Nrg1* is a direct transcriptional target of p63 in basal cells of the mammary gland [151]. As Nrg1 is required for the activation of ERBB4/STAT5A in the luminal cells and thus for luminal progenitor cell maturation, loss of p63 in the basal cells results in defects in luminal cell proliferation and differentiation and consequently in lactation failure during pregnancy through defects in paracrine cell-cell signaling. Further research on p63′s role in epithelial development will certainly increase our understanding of the molecular mechanisms of normal epithelial development, as well as of its role as an oncogene.

## Figures and Tables

**Table 1:** Overview of the most common characteristics seen in patients suffering from different syndromes caused by mutations in p63. +++: Frequent; +: Occasional; -: Rare or absent; *: Skin erosion; ^#^: Excessive freckling; ^1^: Absence of ovaries for mutation 1576–1577DelTT and R604* [120,121]; ^2^: Premature menopause for mutation 1783DelC [122]. See also [112].

		EEC	LMS	ADULT	AEC	RHS	SHFM4	nsOFC
**Limb Defects**	Ectrodactyly	+++	+++	+++	-	-	+++	-
	Syndactyly	+++	+++	+++	+	+	+++	-
**Orofacial Clefting**	Cleft Lip	+++	-	-	+++	+++	-	+++
	Cleft Palate	+++	+++	-	+++	+++	-	+++
**Ectodermal Dysplasia**	Skin	+++	+	+++ ^(#)^	+++ ^(^*^)^	+++	-	-
	Hair	+++	+	+++	+++	+++	-	-
	Nails	+++	+++	+++	+++	+++	-	-
	Teeth	+++	+++	+++	+++	+++	-	-
	Lacrimal Duct	+++	+++	+++	+++	+++	-	-
	Mammary Gland/Nipple	+	+++	+++	+	+	-	-
	Hypohydrosis	+	+++	+	+	+++	-	-
	Ankyloblepharon	-	-	-	+++	+	-	-
	Hearing Impairment	+	+	-	+++	+	-	-
	Genitourinary/Kidney	+	+++ ^(1)^	-	+++	+ ^(2)^	-	-
	Limbal Stem Cell Deficiency	+++	-	-	-	-	-	-

EEC (Ectrodactyly-ectodermal dysplasia-clefting), LMS (Limb mammary syndrome), ADULT (Acro-Dermato-Ungual-Lacrimal-Tooth malformations), AEC (Ankyloblepharon-ectodermal defects-cleft lip/palate), RHS (Rapp-Hodgkin syndrome), SHFM4 (split-hand/split-foot malformations), nsOFC (non-syndromic orofacial clefting).

## Data Availability

Not applicable.

## References

[1] Levine A.J. (2020). p53: 800 million years of evolution and 40 years of discovery. Nat. Rev. Cancer.

[2] Haupt Y., Maya R., Kazaz A., Oren M. (1997). Mdm2 promotes the rapid degradation of p53. Nature.

[3] Kubbutat M.H., Jones S.N., Vousden K.H. (1997). Regulation of p53 stability by Mdm2. Nature.

[4] Kussie P.H., Gorina S., Marechal V., Elenbaas B., Moreau J., Levine A.J., Pavletich N.P. (1996). Structure of the MDM2 oncoprotein bound to the p53 tumor suppressor transactivation domain. Science.

[5] Vousden K.H., Lu X. (2002). Live or let die: The cell’s response to p53. Nat. Rev. Cancer.

[6] Vousden K.H., Prives C. (2009). Blinded by the light: The growing complexity of p53. Cell.

[7] Bieging K.T., Attardi L.D. (2012). Deconstructing p53 transcriptional networks in tumor suppression. Trends Cell Biol..

[8] Vogelstein B., Lane D., Levine A.J. (2000). Surfing the p53 network. Nature.

[9] Riley T., Sontag E., Chen P., Levine A. (2008). Transcriptional control of human p53-regulated genes. Nat. Rev..

[10] Kenzelmann Broz D., Spano Mello S., Bieging K.T., Jiang D., Dusek R.L., Brady C.A., Sidow A., Attardi L.D. (2013). Global genomic profiling reveals an extensive p53-regulated autophagy program contributing to key p53 responses. Genes Dev..

[11] Hollstein M., Sidransky D., Vogelstein B., Harris C.C. (1991). p53 mutations in human cancers. Science.

[12] Freed-Pastor W.A., Prives C. (2012). Mutant p53: One name, many proteins. Genes Dev..

[13] Lane D.P. (1992). Cancer. p53, guardian of the genome. Nature.

[14] Yang A., Kaghad M., Wang Y., Gillett E., Fleming M.D., Dotsch V., Andrews N.C., Caput D., McKeon F. (1998). p63, a p53 homolog at 3q27-29, encodes multiple products with transactivating, death-inducing, and dominant-negative activities. Mol. Cell.

[15] Kaghad M., Bonnet H., Yang A., Creancier L., Biscan J.C., Valent A., Minty A., Chalon P., Lelias J.M., Dumont X. (1997). Monoallelically expressed gene related to p53 at 1p36, a region frequently deleted in neuroblastoma and other human cancers. Cell.

[16] Yang A., Walker N., Bronson R., Kaghad M., Oosterwegel M., Bonnin J., Vagner C., Bonnet H., Dikkes P., Sharpe A. (2000). p73-deficient mice have neurological, pheromonal and inflammatory defects but lack spontaneous tumours. Nature.

[17] Armstrong J.F., Kaufman M.H., Harrison D.J., Clarke A.R. (1995). High-frequency developmental abnormalities in p53-deficient mice. Curr. Biol..

[18] Donehower L.A., Harvey M., Slagle B.L., McArthur M.J., Montgomery C.A., Butel J.S., Bradley A. (1992). Mice deficient for p53 are developmentally normal but susceptible to spontaneous tumours. Nature.

[19] Holembowski L., Kramer D., Riedel D., Sordella R., Nemajerova A., Dobbelstein M., Moll U.M. (2014). TAp73 is essential for germ cell adhesion and maturation in testis. J. Cell Biol..

[20] Inoue S., Tomasini R., Rufini A., Elia A.J., Agostini M., Amelio I., Cescon D., Dinsdale D., Zhou L., Harris I.S. (2014). TAp73 is required for spermatogenesis and the maintenance of male fertility. Proc. Natl. Acad. Sci. USA.

[21] Marshall C.B., Mays D.J., Beeler J.S., Rosenbluth J.M., Boyd K.L., Santos Guasch G.L., Shaver T.M., Tang L.J., Liu Q., Shyr Y. (2016). p73 is Required for multiciliogenesis and regulates the Foxj1-associated gene network. Cell Rep..

[22] Yang A., Schweitzer R., Sun D., Kaghad M., Walker N., Bronson R.T., Tabin C., Sharpe A., Caput D., Crum C. (1999). p63 is essential for regenerative proliferation in limb, craniofacial and epithelial development. Nature.

[23] Mills A.A., Zheng B., Wang X.J., Vogel H., Roop D.R., Bradley A. (1999). p63 is a p53 homologue required for limb and epidermal morphogenesis. Nature.

[24] Senoo M., Pinto F., Crum C.P., McKeon F. (2007). p63 Is essential for the proliferative potential of stem cells in stratified epithelia. Cell.

[25] Suh E.K., Yang A., Kettenbach A., Bamberger C., Michaelis A.H., Zhu Z., Elvin J.A., Bronson R.T., Crum C.P., McKeon F. (2006). p63 protects the female germ line during meiotic arrest. Nature.

[26] Livera G., Petre-Lazar B., Guerquin M.J., Trautmann E., Coffigny H., Habert R. (2008). p63 null mutation protects mouse oocytes from radio-induced apoptosis. Reproduction.

[27] Schumacher B., Hofmann K., Boulton S., Gartner A. (2001). The *C. elegans* homolog of the p53 tumor suppressor is required for DNA damage-induced apoptosis. Curr. Biol..

[28] Derry W.B., Putzke A.P., Rothman J.H. (2001). Caenorhabditis elegans p53: Role in apoptosis, meiosis, and stress resistance. Science.

[29] Ollmann M., Young L.M., Di Como C.J., Karim F., Belvin M., Robertson S., Whittaker K., Demsky M., Fisher W.W., Buchman A. (2000). Drosophila p53 is a structural and functional homolog of the tumor suppressor p53. Cell.

[30] Brodsky M.H., Nordstrom W., Tsang G., Kwan E., Rubin G.M., Abrams J.M. (2000). Drosophila p53 binds a damage response element at the reaper locus. Cell.

[31] Heering J., Jonker H.R., Lohr F., Schwalbe H., Dotsch V. (2016). Structural investigations of the p53/p73 homologs from the tunicate species Ciona intestinalis reveal the sequence requirements for the formation of a tetramerization domain. Protein Sci..

[32] Belyi V.A., Ak P., Markert E., Wang H., Hu W., Puzio-Kuter A., Levine A.J. (2010). The origins and evolution of the p53 family of genes. Cold Spring Harb. Perspect. Biol..

[33] Ou H.D., Lohr F., Vogel V., Mantele W., Dotsch V. (2007). Structural evolution of C-terminal domains in the p53 family. EMBO J..

[34] Gebel J., Tuppi M., Sanger N., Schumacher B., Dotsch V. (2020). DNA Damaged Induced Cell Death in Oocytes. Molecules.

[35] Keeney S., Giroux C.N., Kleckner N. (1997). Meiosis-specific DNA double-strand breaks are catalyzed by Spo11, a member of a widely conserved protein family. Cell.

[36] Romanienko P.J., Camerini-Otero R.D. (2000). The mouse Spo11 gene is required for meiotic chromosome synapsis. Mol. Cell.

[37] Claeys Bouuaert C., Tischfield S.E., Pu S., Mimitou E.P., Arias-Palomo E., Berger J.M., Keeney S. (2021). Structural and functional characterization of the Spo11 core complex. Nat. Struct. Mol. Biol..

[38] Kerr J.B., Hutt K.J., Michalak E.M., Cook M., Vandenberg C.J., Liew S.H., Bouillet P., Mills A., Scott C.L., Findlay J.K. (2012). DNA damage-induced primordial follicle oocyte apoptosis and loss of fertility require TAp63-mediated induction of Puma and Noxa. Mol. Cell.

[39] Deutsch G.B., Zielonka E.M., Coutandin D., Weber T.A., Schafer B., Hannewald J., Luh L.M., Durst F.G., Ibrahim M., Hoffmann J. (2011). DNA damage in oocytes induces a switch of the quality control factor TAp63alpha from dimer to tetramer. Cell.

[40] Coutandin D., Osterburg C., Srivastav R.K., Sumyk M., Kehrloesser S., Gebel J., Tuppi M., Hannewald J., Schafer B., Salah E. (2016). Quality control in oocytes by p63 is based on a spring-loaded activation mechanism on the molecular and cellular level. eLife.

[41] Bolcun-Filas E., Rinaldi V.D., White M.E., Schimenti J.C. (2014). Reversal of female infertility by Chk2 ablation reveals the oocyte DNA damage checkpoint pathway. Science.

[42] Tuppi M., Kehrloesser S., Coutandin D.W., Rossi V., Luh L.M., Strubel A., Hotte K., Hoffmeister M., Schafer B., De Oliveira T. (2018). Oocyte DNA damage quality control requires consecutive interplay of CHK2 and CK1 to activate p63. Nat. Struct. Mol. Biol..

[43] Gebel J., Tuppi M., Chaikuad A., Hotte K., Schroder M., Schulz L., Lohr F., Gutfreund N., Finke F., Henrich E. (2020). p63 uses a switch-like mechanism to set the threshold for induction of apoptosis. Nat. Chem. Biol..

[44] Woodard T.L., Bolcun-Filas E. (2016). Prolonging reproductive life after cancer: The need for fertoprotective therapies. Trends Cancer.

[45] Spears N., Lopes F., Stefansdottir A., Rossi V., De Felici M., Anderson R.A., Klinger F.G. (2019). Ovarian damage from chemotherapy and current approaches to its protection. Hum. Reprod. Update.

[46] Hao X., Anastacio A., Liu K., Rodriguez-Wallberg K.A. (2019). Ovarian follicle depletion induced by chemotherapy and the investigational stages of potential fertility-protective treatments—A review. Int. J. Mol. Sci..

[47] Jeruss J.S., Woodruff T.K. (2009). Preservation of fertility in patients with cancer. N. Engl. J. Med..

[48] Johnston R.J., Wallace W.H. (2009). Normal ovarian function and assessment of ovarian reserve in the survivor of childhood cancer. Pediatr. Blood Cancer.

[49] Maltaris T., Weigel M., Mueller A., Schmidt M., Seufert R., Fischl F., Koelbl H., Dittrich R. (2008). Cancer and fertility preservation: Fertility preservation in breast cancer patients. Breast Cancer Res..

[50] Lena A.M., Rossi V., Osterburg S., Smirnov A., Osterburg C., Tuppi M., Cappello A., Amelio I., Dotsch V., De Felici M. (2021). The p63 C-terminus is essential for murine oocyte integrity. Nat. Commun..

[51] Serber Z., Lai H.C., Yang A., Ou H.D., Sigal M.S., Kelly A.E., Darimont B.D., Duijf P.H., Van Bokhoven H., McKeon F. (2002). A C-terminal inhibitory domain controls the activity of p63 by an intramolecular mechanism. Mol. Cell Biol..

[52] Romano R.A., Smalley K., Magraw C., Serna V.A., Kurita T., Raghavan S., Sinha S. (2012). DeltaNp63 knockout mice reveal its indispensable role as a master regulator of epithelial development and differentiation. Development.

[53] Candi E., Rufini A., Terrinoni A., Dinsdale D., Ranalli M., Paradisi A., De Laurenzi V., Spagnoli L.G., Catani M.V., Ramadan S. (2006). Differential roles of p63 isoforms in epidermal development: Selective genetic complementation in p63 null mice. Cell Death Differ..

[54] Krauskopf K., Gebel J., Kazemi S., Tuppi M., Lohr F., Schafer B., Koch J., Guntert P., Dotsch V., Kehrloesser S. (2018). Regulation of the activity in the p53 family depends on the organization of the transactivation domain. Structure.

[55] Krois A.S., Ferreon J.C., Martinez-Yamout M.A., Dyson H.J., Wright P.E. (2016). Recognition of the disordered p53 transactivation domain by the transcriptional adapter zinc finger domains of CREB-binding protein. Proc. Natl. Acad. Sci. USA.

[56] Burge S., Teufel D.P., Townsley F.M., Freund S.M., Bycroft M., Fersht A.R. (2009). Molecular basis of the interactions between the p73 N terminus and p300: Effects on transactivation and modulation by phosphorylation. Proc. Natl. Acad. Sci. USA.

[57] Yang A., Zhu Z., Kettenbach A., Kapranov P., McKeon F., Gingeras T.R., Struhl K. (2010). Genome-wide mapping indicates that p73 and p63 co-occupy target sites and have similar dna-binding profiles in vivo. PLoS ONE.

[58] Carroll D.K., Carroll J.S., Leong C.O., Cheng F., Brown M., Mills A.A., Brugge J.S., Ellisen L.W. (2006). p63 regulates an adhesion programme and cell survival in epithelial cells. Nat. Cell Biol..

[59] Truong A.B., Kretz M., Ridky T.W., Kimmel R., Khavari P.A. (2006). p63 regulates proliferation and differentiation of developmentally mature keratinocytes. Genes Dev..

[60] Soares E., Zhou H. (2018). Master regulatory role of p63 in epidermal development and disease. Cell Mol. Life Sci..

[61] Kouwenhoven E.N., Oti M., Niehues H., van Heeringen S.J., Schalkwijk J., Stunnenberg H.G., van Bokhoven H., Zhou H. (2015). Transcription factor p63 bookmarks and regulates dynamic enhancers during epidermal differentiation. EMBO Rep..

[62] Qu J., Tanis S.E.J., Smits J.P.H., Kouwenhoven E.N., Oti M., van den Bogaard E.H., Logie C., Stunnenberg H.G., van Bokhoven H., Mulder K.W. (2018). Mutant p63 affects epidermal cell identity through rewiring the enhancer landscape. Cell Rep..

[63] Soares E., Xu Q., Li Q., Qu J., Zheng Y., Raeven H.H.M., Brandao K.O., Petit I., van den Akker W.M.R., van Heeringen S.J. (2019). Single-cell RNA-seq identifies a reversible mesodermal activation in abnormally specified epithelia of p63 EEC syndrome. Proc. Natl. Acad. Sci. USA.

[64] Gonzales K.A.U., Fuchs E. (2017). Skin and its regenerative powers: An alliance between stem cells and their niche. Dev. Cell.

[65] Sada A., Jacob F., Leung E., Wang S., White B.S., Shalloway D., Tumbar T. (2016). Defining the cellular lineage hierarchy in the interfollicular epidermis of adult skin. Nat. Cell Biol..

[66] Tickle C. (2003). Patterning systems—From one end of the limb to the other. Dev. Cell.

[67] Niswander L. (2002). Interplay between the molecular signals that control vertebrate limb development. Int. J. Dev. Biol..

[68] Coutandin D., Lohr F., Niesen F.H., Ikeya T., Weber T.A., Schafer B., Zielonka E.M., Bullock A.N., Yang A., Guntert P. (2009). Conformational stability and activity of p73 require a second helix in the tetramerization domain. Cell Death Differ..

[69] Joerger A.C., Rajagopalan S., Natan E., Veprintsev D.B., Robinson C.V., Fersht A.R. (2009). Structural evolution of p53, p63, and p73: Implication for heterotetramer formation. Proc. Natl. Acad. Sci. USA.

[70] Gebel J., Luh L.M., Coutandin D., Osterburg C., Lohr F., Schafer B., Frombach A.S., Sumyk M., Buchner L., Krojer T. (2016). Mechanism of TAp73 inhibition by DeltaNp63 and structural basis of p63/p73 hetero-tetramerization. Cell Death Differ..

[71] Beeler J.S., Marshall C.B., Gonzalez-Ericsson P.I., Shaver T.M., Santos Guasch G.L., Lea S.T., Johnson K.N., Jin H., Venters B.J., Sanders M.E. (2019). p73 regulates epidermal wound healing and induced keratinocyte programming. PLoS ONE.

[72] Ferone G., Mollo M.R., Missero C. (2015). Epidermal cell junctions and their regulation by p63 in health and disease. Cell Tissue Res..

[73] Ferone G., Mollo M.R., Thomason H.A., Antonini D., Zhou H.Q., Ambrosio R., De Rosa L., Salvatore D., Getsios S., van Bokhoven H. (2013). p63 control of desmosome gene expression and adhesion is compromised in AEC syndrome. Hum. Mol. Genet..

[74] Nguyen B.C., Lefort K., Mandinova A., Antonini D., Devgan V., Della Gatta G., Koster M.I., Zhang Z., Wang J., di Vignano A.T. (2006). Cross-regulation between Notch and p63 in keratinocyte commitment to differentiation. Gene Dev..

[75] Romano R.A., Birkaya B., Sinha S. (2007). A functional enhancer of keratin14 is a direct transcriptional target of deltaNp63. J. Investig. Dermatol..

[76] Lopardo T., Lo Iacono N., Marinari B., Giustizieri M.L., Cyr D.G., Merlo G., Crosti F., Costanzo A., Guerrini L. (2008). Claudin-1 is a p63 target gene with a crucial role in epithelial development. PLoS ONE.

[77] Ihrie R.A., Marques M.R., Nguyen B.T., Horner J.S., Papazoglu C., Bronson R.T., Mills A.A., Attardi L.D. (2005). Perp is a p63-regulated gene essential for epithelial integrity. Cell.

[78] Kouwenhoven E.N., van Bokhoven H., Zhou H. (2015). Gene regulatory mechanisms orchestrated by p63 in epithelial development and related disorders. Biochim. Biophys. Acta.

[79] Sethi I., Gluck C., Zhou H., Buck M.J., Sinha S. (2017). Evolutionary re-wiring of p63 and the epigenomic regulatory landscape in keratinocytes and its potential implications on species-specific gene expression and phenotypes. Nucl. Acids Res..

[80] Kouwenhoven E.N., van Heeringen S.J., Tena J.J., Oti M., Dutilh B.E., Alonso M.E., de la Calle-Mustienes E., Smeenk L., Rinne T., Parsaulian L. (2010). Genome-wide profiling of p63 DNA-binding sites identifies an element that regulates gene expression during limb development in the 7q21 SHFM1 locus. PLoS Genet..

[81] McDade S.S., Henry A.E., Pivato G.P., Kozarewa I., Mitsopoulos C., Fenwick K., Assiotis I., Hakas J., Zvelebil M., Orr N. (2012). Genome-wide analysis of p63 binding sites identifies AP-2 factors as co-regulators of epidermal differentiation. Nucl. Acids Res..

[82] Rapisarda V., Malashchuk I., Asamaowei I.E., Poterlowicz K., Fessing M.Y., Sharov A.A., Karakesisoglou I., Botchkarev V.A., Mardaryev A. (2017). p63 transcription factor regulates nuclear shape and Expression of nuclear envelope-associated genes in epidermal keratinocytes. J. Investig. Dermatol..

[83] Ramsey M.R., He L., Forster N., Ory B., Ellisen L.W. (2011). Physical association of HDAC1 and HDAC2 with p63 mediates transcriptional repression and tumor maintenance in squamous cell carcinoma. Cancer Res..

[84] Qu J., Yi G., Zhou H. (2019). p63 cooperates with CTCF to modulate chromatin architecture in skin keratinocytes. Epigenet. Chrom..

[85] LeBoeuf M., Terrell A., Trivedi S., Sinha S., Epstein J.A., Olson E.N., Morrisey E.E., Millar S.E. (2010). Hdac1 and Hdac2 act redundantly to control p63 and p53 functions in epidermal progenitor cells. Dev. Cell.

[86] Westfall M.D., Mays D.J., Sniezek J.C., Pietenpol J.A. (2003). The Delta Np63 alpha phosphoprotein binds the p21 and 14-3-3 sigma promoters in vivo and has transcriptional repressor activity that is reduced by Hay-Wells syndrome-derived mutations. Mol. Cell Biol..

[87] Fan X.Y., Wang D.M., Burgmaier J.E., Teng Y.D., Romano R.A., Sinha S., Yi R. (2018). Single cell and open chromatin analysis reveals molecular origin of epidermal cells of the skin. Dev. Cell.

[88] Cavazza A., Miccio A., Romano O., Petiti L., Malagoli Tagliazucchi G., Peano C., Severgnini M., Rizzi E., De Bellis G., Bicciato S. (2016). Dynamic Transcriptional and Epigenetic Regulation of Human Epidermal Keratinocyte Differentiation. Stem Cell Rep..

[89] Hnisz D., Abraham B.J., Lee T.I., Lau A., Saint-Andre V., Sigova A.A., Hoke H.A., Young R.A. (2013). Super-enhancers in the control of cell identity and disease. Cell.

[90] Pasquali L., Gaulton K.J., Rodriguez-Segui S.A., Mularoni L., Miguel-Escalada I., Akerman I., Tena J.J., Moran I., Gomez-Marin C., van de Bunt M. (2014). Pancreatic islet enhancer clusters enriched in type 2 diabetes risk-associated variants. Nat. Genet..

[91] Rinaldi L., Datta D., Serrat J., Morey L., Solanas G., Avgustinova A., Blanco E., Pons J.I., Matallanas D., Von Kriegsheim A. (2016). Dnmt3a and Dnmt3b associate with enhancers to regulate human epidermal stem cell homeostasis. Cell Stem Cell.

[92] Li L., Wang Y., Torkelson J.L., Shankar G., Pattison J.M., Zhen H.H., Fang F., Duren Z., Xin J., Gaddam S. (2019). TFAP2C- and p63-dependent networks sequentially rearrange chromatin landscapes to drive human epidermal lineage commitment. Cell Stem Cell.

[93] Yang A., Zhu Z., Kapranov P., McKeon F., Church G.M., Gingeras T.R., Struhl K. (2006). Relationships between p63 binding, DNA sequence, transcription activity, and biological function in human cells. Mol. Cell.

[94] Sethi I., Sinha S., Buck M.J. (2014). Role of chromatin and transcriptional co-regulators in mediating p63-genome interactions in keratinocytes. BMC Genom..

[95] Chen Y., Mistry D.S., Sen G.L. (2014). Highly rapid and efficient conversion of human fibroblasts to keratinocyte-like cells. J. Investig. Dermatol..

[96] Russo C., Osterburg C., Sirico A., Antonini D., Ambrosio R., Wurz J.M., Rinnenthal J., Ferniani M., Kehrloesser S., Schafer B. (2018). Protein aggregation of the p63 transcription factor underlies severe skin fragility in AEC syndrome. Proc. Natl. Acad. Sci. USA.

[97] Ouyang H., Xue Y., Lin Y., Zhang X., Xi L., Patel S., Cai H., Luo J., Zhang M., Zhang M. (2014). WNT7A and PAX6 define corneal epithelium homeostasis and pathogenesis. Nature.

[98] Di Iorio E., Kaye S.B., Ponzin D., Barbaro V., Ferrari S., Bohm E., Nardiello P., Castaldo G., McGrath J.A., Willoughby C.E. (2012). Limbal stem cell deficiency and ocular phenotype in ectrodactyly-ectodermal dysplasia-clefting syndrome caused by p63 mutations. Ophthalmology.

[99] Secker G.A., Daniels J.T. (2008). Corneal epithelial stem cells: Deficiency and regulation. Stem Cell Rev..

[100] Fessing M.Y., Mardaryev A.N., Gdula M.R., Sharov A.A., Sharova T.Y., Rapisarda V., Gordon K.B., Smorodchenko A.D., Poterlowicz K., Ferone G. (2011). p63 regulates Satb1 to control tissue-specific chromatin remodeling during development of the epidermis. J. Cell Biol..

[101] Mardaryev A.N., Liu B., Rapisarda V., Poterlowicz K., Malashchuk I., Rudolf J., Sharov A.A., Jahoda C.A., Fessing M.Y., Benitah S.A. (2016). Cbx4 maintains the epithelial lineage identity and cell proliferation in the developing stratified epithelium. J. Cell Biol..

[102] Mardaryev A.N., Gdula M.R., Yarker J.L., Emelianov V.U., Poterlowicz K., Sharov A.A., Sharova T.Y., Scarpa J.A., Joffe B., Solovei I. (2014). p63 and Brg1 control developmentally regulated higher-order chromatin remodelling at the epidermal differentiation complex locus in epidermal progenitor cells. Development.

[103] Keyes W.M., Pecoraro M., Aranda V., Vernersson-Lindahl E., Li W., Vogel H., Guo X., Garcia E.L., Michurina T.V., Enikolopov G. (2011). DeltaNp63alpha is an oncogene that targets chromatin remodeler Lsh to drive skin stem cell proliferation and tumorigenesis. Cell Stem Cell.

[104] Straub W.E., Weber T.A., Schafer B., Candi E., Durst F., Ou H.D., Rajalingam K., Melino G., Dotsch V. (2010). The C-terminus of p63 contains multiple regulatory elements with different functions. Cell Death Dis..

[105] Sammons M.A., Zhu J.J., Drake A.M., Berger S.L. (2015). TP53 engagement with the genome occurs in distinct local chromatin environments via pioneer factor activity. Genome Res..

[106] Bao X., Rubin A.J., Qu K., Zhang J., Giresi P.G., Chang H.Y., Khavari P.A. (2015). A novel ATAC-seq approach reveals lineage-specific reinforcement of the open chromatin landscape via cooperation between BAF and p63. Genome Biol..

[107] Santos-Pereira J.M., Gallardo-Fuentes L., Neto A., Acemel R.D., Tena J.J. (2019). Pioneer and repressive functions of p63 during zebrafish embryonic ectoderm specification. Nat. Commun..

[108] Celli J., Duijf P., Hamel B.C., Bamshad M., Kramer B., Smits A.P., Newbury-Ecob R., Hennekam R.C., Van Buggenhout G., van Haeringen A. (1999). Heterozygous germline mutations in the p53 homolog p63 are the cause of EEC syndrome. Cell.

[109] Duijf P.H., van Bokhoven H., Brunner H.G. (2003). Pathogenesis of split-hand/split-foot malformation. Hum. Mol. Genet..

[110] McGrath J.A., Duijf P.H., Doetsch V., Irvine A.D., de Waal R., Vanmolkot K.R., Wessagowit V., Kelly A., Atherton D.J., Griffiths W.A. (2001). Hay-Wells syndrome is caused by heterozygous missense mutations in the SAM domain of p63. Hum. Mol. Genet..

[111] Rinne T., Bolat E., Meijer R., Scheffer H., van Bokhoven H. (2009). Spectrum of p63 mutations in a selected patient cohort affected with ankyloblepharon-ectodermal defects-cleft lip/palate syndrome (AEC). Am. J. Med. Genet..

[112] Rinne T., Hamel B., van Bokhoven H., Brunner H.G. (2006). Pattern of p63 mutations and their phenotypes—Update. Am. J. Med. Genet..

[113] Vernersson Lindahl E., Garcia E.L., Mills A.A. (2013). An allelic series of Trp63 mutations defines TAp63 as a modifier of EEC syndrome. Am. J. Med. Genet..

[114] Prontera P., Garelli E., Isidori I., Mencarelli A., Carando A., Silengo M.C., Donti E. (2011). Cleft palate and ADULT phenotype in a patient with a novel TP63 mutation suggests lumping of EEC/LM/ADULT syndromes into a unique entity: ELA syndrome. Am. J. Med. Genet..

[115] Maillard A., Alby C., Gabison E., Doan S., Caux F., Bodemer C., Hadj-Rabia S. (2019). P63-related disorders: Dermatological characteristics in 22 patients. Exp. Dermatol.

[116] van Bokhoven H., Hamel B.C., Bamshad M., Sangiorgi E., Gurrieri F., Duijf P.H., Vanmolkot K.R., van Beusekom E., van Beersum S.E., Celli J. (2001). p63 Gene mutations in eec syndrome, limb-mammary syndrome, and isolated split hand-split foot malformation suggest a genotype-phenotype correlation. Am. J. Hum. Genet..

[117] Leoyklang P., Siriwan P., Shotelersuk V. (2006). A mutation of the p63 gene in non-syndromic cleft lip. J. Med. Genet..

[118] Chitayat D., Babul R., Silver M.M., Jay V., Teshima I.E., Babyn P., Becker L.E. (1996). Terminal deletion of the long arm of chromosome 3 [46,XX,del(3)(q27-->qter)]. Am. J. Med. Genet..

[119] Khandelwal K.D., van den Boogaard M.H., Mehrem S.L., Gebel J., Fagerberg C., van Beusekom E., van Binsbergen E., Topaloglu O., Steehouwer M., Gilissen C. (2019). Deletions and loss-of-function variants in TP63 associated with orofacial clefting. Eur. J. Hum. Genet..

[120] Guazzarotti L., Caprio C., Rinne T.K., Bosoni M., Pattarino G., Mauri S., Tadini G.L., van Bokhoven H., Zuccotti G.V. (2008). Limb-mammary syndrome (LMS) associated with internal female genitalia dysgenesia: A new genotype/phenotype correlation?. Am. J. Med. Genet..

[121] Mathorne S.W., Ravn P., Hansen D., Beck-Nielsen S.S., Gjorup H., Sorensen K.P., Fagerberg C.R. (2020). Novel phenotype of syndromic premature ovarian insufficiency associated with TP63 molecular defect. Clin. Genet..

[122] Holder-Espinasse M., Martin-Coignard D., Escande F., Manouvrier-Hanu S. (2007). A new mutation in TP63 is associated with age-related pathology. Eur. J. Hum. Genet..

[123] Browne G., Cipollone R., Lena A.M., Serra V., Zhou H., van Bokhoven H., Dotsch V., Merico D., Mantovani R., Terrinoni A. (2011). Differential altered stability and transcriptional activity of DeltaNp63 mutants in distinct ectodermal dysplasias. J. Cell Sci..

[124] Della Gatta G., Bansal M., Ambesi-Impiombato A., Antonini D., Missero C., di Bernardo D. (2008). Direct targets of the TRP63 transcription factor revealed by a combination of gene expression profiling and reverse engineering. Genome Res..

[125] Shen J., van den Bogaard E.H., Kouwenhoven E.N., Bykov V.J., Rinne T., Zhang Q., Tjabringa G.S., Gilissen C., van Heeringen S.J., Schalkwijk J. (2013). APR-246/PRIMA-1(MET) rescues epidermal differentiation in skin keratinocytes derived from EEC syndrome patients with p63 mutations. Proc. Natl. Acad. Sci. USA.

[126] Lo Iacono N., Mantero S., Chiarelli A., Garcia E., Mills A.A., Morasso M.I., Costanzo A., Levi G., Guerrini L., Merlo G.R. (2008). Regulation of Dlx5 and Dlx6 gene expression by p63 is involved in EEC and SHFM congenital limb defects. Development.

[127] Merlo G.R., Zerega B., Paleari L., Trombino S., Mantero S., Levi G. (2000). Multiple functions of Dlx genes. Int. J. Dev. Biol..

[128] Merlo G.R., Paleari L., Mantero S., Genova F., Beverdam A., Palmisano G.L., Barbieri O., Levi G. (2002). Mouse model of split hand/foot malformation type I. Genesis.

[129] Acampora D., Merlo G.R., Paleari L., Zerega B., Postiglione M.P., Mantero S., Bober E., Barbieri O., Simeone A., Levi G. (1999). Craniofacial, vestibular and bone defects in mice lacking the Distal-less-related gene Dlx5. Development.

[130] Bakkers J., Hild M., Kramer C., Furutani-Seiki M., Hammerschmidt M. (2002). Zebrafish DeltaNp63 is a direct target of Bmp signaling and encodes a transcriptional repressor blocking neural specification in the ventral ectoderm. Dev. Cell.

[131] Ferone G., Thomason H.A., Antonini D., De Rosa L., Hu B., Gemei M., Zhou H., Ambrosio R., Rice D.P., Acampora D. (2012). Mutant p63 causes defective expansion of ectodermal progenitor cells and impaired FGF signalling in AEC syndrome. EMBO Mol. Med..

[132] Zarnegar B.J., Webster D.E., Lopez-Pajares V., Vander Stoep Hunt B., Qu K., Yan K.J., Berk D.R., Sen G.L., Khavari P.A. (2012). Genomic profiling of a human organotypic model of AEC syndrome reveals ZNF750 as an essential downstream target of mutant TP63. Am. J. Hum. Genet..

[133] Sen G.L., Boxer L.D., Webster D.E., Bussat R.T., Qu K., Zarnegar B.J., Johnston D., Siprashvili Z., Khavari P.A. (2012). ZNF750 is a p63 target gene that induces KLF4 to drive terminal epidermal differentiation. Dev. Cell.

[134] Lorch J.H., Klessner J., Park J.K., Getsios S., Wu Y.L., Stack M.S., Green K.J. (2004). Epidermal growth factor receptor inhibition promotes desmosome assembly and strengthens intercellular adhesion in squamous cell carcinoma cells. J. Biol. Chem..

[135] Yin T.F., Getsios S., Caldelari R., Godsel L.M., Kowalczyk A.P., Muller E.J., Green K.J. (2005). Mechanisms of plakoglobin-dependent adhesion—Desmosome-specific functions in assembly and regulation by epidermal growth factor receptor. J. Biol. Chem..

[136] Sathyamurthy A., Freund S.M., Johnson C.M., Allen M.D., Bycroft M. (2011). Structural basis of p63alpha SAM domain mutants involved in AEC syndrome. FEBS J..

[137] Levine A.J., Tomasini R., McKeon F.D., Mak T.W., Melino G. (2011). The p53 family: Guardians of maternal reproduction. Nat. Rev..

[138] Gebel J., Tuppi M., Krauskopf K., Coutandin D., Pitzius S., Kehrloesser S., Osterburg C., Dotsch V. (2017). Control mechanisms in germ cells mediated by p53 family proteins. J. Cell Sci..

[139] Tucker E.J., Grover S.R., Robevska G., van den Bergen J., Hanna C., Sinclair A.H. (2018). Identification of variants in pleiotropic genes causing “isolated” premature ovarian insufficiency: Implications for medical practice. Eur. J. Hum. Genet..

[140] Tucker E.J., Jaillard S., Grover S.R., van den Bergen J., Robevska G., Bell K.M., Sadedin S., Hanna C., Dulon J., Touraine P. (2019). TP63-truncating variants cause isolated premature ovarian insufficiency. Hum. Mutat..

[141] Bestetti I., Castronovo C., Sironi A., Caslini C., Sala C., Rossetti R., Crippa M., Ferrari I., Pistocchi A., Toniolo D. (2019). High-resolution array-CGH analysis on 46,XX patients affected by early onset primary ovarian insufficiency discloses new genes involved in ovarian function. Hum. Reprod..

[142] Keyes W.M., Vogel H., Koster M.I., Guo X., Qi Y., Petherbridge K.M., Roop D.R., Bradley A., Mills A.A. (2006). p63 heterozygous mutant mice are not prone to spontaneous or chemically induced tumors. Proc. Natl. Acad. Sci. USA.

[143] Ramsey M.R., Wilson C., Ory B., Rothenberg S.M., Faquin W., Mills A.A., Ellisen L.W. (2013). FGFR2 signaling underlies p63 oncogenic function in squamous cell carcinoma. J. Clin. Investig..

[144] Rocco J.W., Leong C.O., Kuperwasser N., DeYoung M.P., Ellisen L.W. (2006). p63 mediates survival in squamous cell carcinoma by suppression of p73-dependent apoptosis. Cancer Cell.

[145] Chakrabarti R., Wei Y., Hwang J., Hang X., Andres Blanco M., Choudhury A., Tiede B., Romano R.A., DeCoste C., Mercatali L. (2014). DeltaNp63 promotes stem cell activity in mammary gland development and basal-like breast cancer by enhancing Fzd7 expression and Wnt signalling. Nat. Cell Biol..

[146] Yalcin-Ozuysal O., Fiche M., Guitierrez M., Wagner K.U., Raffoul W., Brisken C. (2010). Antagonistic roles of Notch and p63 in controlling mammary epithelial cell fates. Cell Death Differ..

[147] Su X.H., Chakravarti D., Cho M.S., Liu L.Z., Gi Y.J., Lin Y.L., Leung M.L., El-Naggar A., Creighton C.J., Suraokar M.B. (2010). TAp63 suppresses metastasis through coordinate regulation of Dicer and miRNAs. Nature.

[148] Stransky N., Egloff A.M., Tward A.D., Kostic A.D., Cibulskis K., Sivachenko A., Kryukov G.V., Lawrence M.S., Sougnez C., McKenna A. (2011). The mutational landscape of head and neck squamous cell carcinoma. Science.

[149] Pitzius S., Osterburg C., Gebel J., Tascher G., Schafer B., Zhou H., Munch C., Dotsch V. (2019). TA*p63 and GTAp63 achieve tighter transcriptional regulation in quality control by converting an inhibitory element into an additional transactivation domain. Cell Death Dis..

[150] Gatti V., Bongiorno-Borbone L., Fierro C., Annicchiarico-Petruzzelli M., Melino G., Peschiaroli A. (2019). p63 at the Crossroads between Stemness and Metastasis in Breast Cancer. Int. J. Mol. Sci..

[151] Forster N., Saladi S.V., van Bragt M., Sfondouris M.E., Jones F.E., Li Z., Ellisen L.W. (2014). Basal cell signaling by p63 controls luminal progenitor function and lactation via NRG1. Dev. Cell.

